# Can cultivated hamster cells compete with chicken meat? Insights on acceptance and digestibility in domestic cats

**DOI:** 10.3389/fvets.2026.1781530

**Published:** 2026-05-21

**Authors:** Federica Bortolazzi, Simone Stringhetti, Myriam Hesta

**Affiliations:** 1 Department of Morphology, Imaging, Orthopedics, Rehabilitation and Nutrition, Faculty of Veterinary Medicine, Ghent University, Ghent, Belgium; 2 Bene Meat Technologies a.s., Prague, Czechia

**Keywords:** acceptance, alternative proteins, cat, cultivated meat, cultured meat, digestibility, hamster, petfood

## Abstract

In response to the challenges posed by a growing human and pet population and the climate crisis, novel ingredients such as cultivated meat have emerged as promising solutions to address concerns related to sustainability, ethics and food security. However, data on its nutritional performance and suitability for use in animal nutrition remain limited. This study aims to evaluate the acceptance and apparent total tract digestibility (AD) of a complete feline diet containing cultivated hamster cell biomass as the sole animal-derived ingredient, compared to a control diet based on chicken breast meat. A two-day, double-blinded, cross-over acceptance test was conducted on 10 adult research cats. Subsequently, eight of these cats were enrolled in a double-blinded, cross-over digestibility trial. Each cat received both the test and control diet for a 7–12 day adaptation period, followed by 5 days of fecal collection. The diets were identical in ingredient composition except for the protein source and were formulated to achieve comparable nutritional profiles. Celite® (acid-insoluble ash) was added as an indigestible marker to calculate AD using an indicator method. Cats were closely monitored for clinical signs. Body weight, body and muscle condition score were assessed weekly, and total food intake was recorded for each meal. Acceptance was optimal in 9 out of 10 cats, with minimal food leftovers. AD results were comparable between diets, except for protein digestibility which was significantly higher in the control diet (85.3 ± 1.6% vs. 83.9 ± 1.2%; *p*-value: 0.033). Interestingly, leftovers were significantly lower for the test diet (2.4 ± 3.3% vs. 8.5 ± 7.8%; *p*-value: 0.025), despite its lower inclusion of palatability enhancer compared to the control diet (1.5 vs. 10 g/kg). Fecal consistency was overall in the ideal range throughout the trial and both diets were well tolerated during the feeding period of up to 17 days. These findings suggest that the cultivated hamster cell biomass tested is well accepted by cats and adequately digestible for use in feline diets. Further research is warranted to explore its impact on palatability and to assess long-term health outcomes in cats.

## 1 Introduction

The global population of dogs (*Canis lupus familiaris*) and cats (*Felis silvestris catus*) continues to rise, fueling demand for pet food, a trend that must be considered in discussions on food system sustainability, due to its significant environmental impact (1–3). Animal-derived proteins have substantially higher carbon, land and water footprints than plant-based ones (4). Consequently, reducing consumption of animal-derived foods has been recommended to mitigate environmental impacts (5), a call that should extend to companion animals where feasible, as pet food contributes considerably to agricultural land use, greenhouse gas emissions, and water consumption (1). The environmental impact of pet food is often assumed negligible because of its reliance on animal by-products (ABPs), yet this assumption is misleading. ABPs represent a significant share of formulations but do not fully meet the sector’s demand for animal-derived ingredients. Although global data are limited, patterns are likely similar across Western countries (1). A 2025 U. S. report based on mid-2023 to mid-2024 data found that meat accounted for 39% of animal ingredients used in pet food, totaling 1.84 million tons, while fish and crustaceans represented 9%, and ABPs (organ meats, broths, rendered meals and fats) comprised 52% (6). Importantly, ABPs are not valueless waste; they are utilized by multiple industries, and their allocation to pet food is primarily driven by market price rather than waste reduction (7). By increasing meat industry profitability, ABPs indirectly reinforce demand for animal farming, thereby contributing to the impact of the sector. Combined with the partial use of meat suitable for human consumption, this dynamic amplifies the influence of pet food demand, ultimately driving additional animal raising and slaughtering (1, 7). Beyond environmental implications, this raises ethical concerns and the so-called “vegetarian dilemma” among non-meat eaters who care for dogs or cats (8), particularly given the existence of welfare issues in animal agriculture (9). Nevertheless, the carnivorous nature of companion animals, especially cats, requires palatable and nutritionally adequate diets, which may be more challenging to achieve when animal-derived ingredients are excluded. Dogs, due to evolutionary adaptations such as enhanced starch utilization, are considered facultative carnivores, whereas cats remain obligate carnivores with strict requirements for nutrients such as taurine, arachidonic acid, and vitamin A, all naturally abundant in animal tissues (10–12).

Several strategies have been proposed to address sustainability and ethical concerns in pet food. Plant-based diets are gaining popularity among pet owners, yet they pose nutritional challenges, particularly for cats. Although synthetic supplementation of essential nutrients such as taurine and arachidonic acid alongside standard vitamins and minerals premixes can theoretically meet feline requirements, some studies have reported nutritional inadequacies in plant-based diets for cats, suggesting that producing nutritionally adequate final products may still be challenging in practice (13, 14). Insects-based diets offer another promising option due to their favorable environmental and nutritional profile (15, 16) and their presence in cats’ natural prey (17). However, ethical concerns regarding insect use and limited knowledge and legislation on insect welfare remain unresolved (18, 19). Additionally, insect nutrient composition varies with rearing conditions and life stage, posing formulation challenges (20, 21). Microbial proteins also show potential to reduce reliance on animal ingredients and mitigate the environmental impact of the meat industry (22). A recent study demonstrated adequate palatability, safety, digestibility, and nutritional adequacy in dogs fed diets containing up to 8% microbial protein, suggesting strong applicability in pet food (23). Nonetheless, limited research and knowledge remain major constraints across all alternative protein sources.

Cultivated meat (CM) represents another promising novel ingredient. Produced by cultivating animal cells under controlled conditions, CM aims to address sustainability, public health, and animal welfare concerns associated with conventional meat production. Compared to other alternatives, CM most closely resembles the natural animal-based diet of carnivorous companion animals. Current evidence indicates that CM can reproduce a nutritional profile broadly comparable to that of conventional meat, although marked variability remains due to differences in cell types, scaffolds (an edible structure used to form the shape and texture of meat), and culture conditions. These findings suggest future potential to closely replicate conventional meat and to fine-tune cultivation parameters for producing targeted nutritional profiles (24, 25). CM also offers several potential advantages over conventionally produced meat, such as reducing farmed animal numbers and antibiotic use and lowering zoonotic disease risk as well as carbon and land footprints (26, 27). Anticipatory life cycle assessments modeling the future of this technology predict environmental benefits compared to conventional meat when powered by renewable energy (28). Adoption of CM in pet diets may occur more rapidly than in human diets due to higher consumer acceptance and fewer regulatory and technological hurdles. A survey reported that 47.3% of respondents were willing to feed CM to their pets, compared to 32.5% willing to consume it themselves (29). Additionally, surveys of dog and cat owners indicate that CM is the preferred alternative protein source for pet food (30, 31). Furthermore, CM for pet food does not require a complex 3D structuring because it is used as an ingredient rather than a whole-cut product; the cell biomass can be directly incorporated into wet or dry formulations, reducing production complexity and cost.

To the author’s knowledge, no studies have yet reported data related to CM consumption in cats and dogs. When introducing a novel ingredient in pet food, it is essential to evaluate both palatability and digestibility. Even if an ingredient meets nutritional requirements, it must first be voluntarily consumed by the animal to provide any benefit. Cats are particularly selective in their feeding behavior and may reject foods with unfamiliar or unpleasant odors, tastes or textures, making palatability a critical factor in diet formulation (32, 33). Furthermore, assessing digestibility is a first important step in evaluating nutrient absorption, as differences in ingredient composition or processing can significantly influence nutrient digestibility and overall diet quality. In the present study, apparent total tract digestibility (AD) was calculated, which is defined as the proportion of an ingested nutrient not recovered in the feces (34). It is termed apparent because it does not distinguish undigested dietary nutrients from endogenous and microbial fecal losses (35). The aim of this study is to compare the acceptance and AD of a complete feline wet diet containing cultivated hamster cell biomass with a control diet formulated using a conventional, highly digestible ingredient such as chicken breast meat. This work provides foundational data that represent an initial step in the evidence base for evaluating this novel ingredient for potential inclusion in pet diets.

## 2 Materials and methods

This double-blinded, cross-over, placebo-controlled study was approved by the Ethical Committee on Animal Experimentation of the Faculties of Veterinary Medicine and Bioscience Engineering at Ghent University (file no. 2025-08).

### 2.1 Animals and housing conditions

Ten healthy research cats (European shorthair; 5 neutered males, 5 spayed females; age: 2 year; body weight: 3.3–5.0 kg; body condition score: 4–6/9; normal muscle condition) were enrolled in a two-day acceptance test. Subsequently, eight cats (4 males, 4 females) were selected for the digestibility trial. Prior to the trial, all cats underwent physical examination and received routine deworming and ectoparasite treatment. The animals were housed in a single group with access to indoor facilities and a fully enclosed outdoor area, including overhead fencing to prevent wildlife access, and were moved to individual enclosures only during meals (up to 2 h per meal, twice daily) and fecal collection period (120 h). During collection periods, a rotation system provided individual daily access to a play area. Each individual enclosure included identification systems, water ad libitum, feeding area, and a secluded section with frosted glass, litter box, and an elevated shelf. Group housing areas also included environmental enrichments in addition to litter boxes and water sources.

### 2.2 Diets

Two diets (test and control) were formulated for this study as complete and balanced wet foods in compliance with European Pet Food Industry Federation (FEDIAF) guidelines (34). Both diets had comparable nutrient profiles and identical ingredient composition, except for the protein source: the test diet contained cultivated hamster cell biomass whereas the control diet contained conventional chicken breast meat. Ingredient composition and inclusion levels (as fed basis) are shown in Table 1. The cultivated cells biomass was included at 24.4% while the chicken meat at 18.2% on a dry matter (DM) basis. Ingredient inclusion levels on DM basis were similar between diets, except for palatability enhancer, which was higher in the control diet to address poor acceptance observed during its development phase. Water addition to the control diet (496.7 vs. 351.6 g/kg) and a slightly adjusted oil inclusion (differing by 0.5 g/kg) were necessary to achieve a similar nutrient profile. Celite® (acid-insoluble ash) was added at 1.5% DM as an indigestible marker for apparent digestibility determination using the indicator method (34). To ensure uniform ingredient distribution, diets were blended to achieve a mousse-like texture. The acceptance test used a preliminary batch of both diets, produced without the premix and indigestible marker, and with a slightly different, more homogeneous texture compared to the batch used in the digestibility trial.

**Table 1:** Ingredients control and test diet (as-fed basis).

Ingredients (g/kg)	CO diet	CM diet
Water	496.7	351.6
Cultivated hamster cells	0	296.3
Chicken breast meat	142.2	0
Chunky grit (pea)	177.7	177.8
Pumpkin	76.0	76.0
Carrot	69.1	69.1
Sunflower oil	11.4	10.9
Linen oil	3.0	3.0
Brewer’s yeast	1.6	1.6
Palatability enhancer	9.9	1.5
Premix	9.9	9.9
Celite	2.6	2.5

CO, conventional meat (control diet); CM, cultivated meat (test diet).

### 2.3 Acceptance test

A two-day, double-blinded, cross-over acceptance test was conducted prior to the digestibility trial to assess voluntary consumption of the diets. Cats were randomly allocated by sex into two groups and received two meals of each diet. The morning meal (131–149 g) was calculated to provide half of the daily energy requirement based on individual historical intake data, while afternoon meals (92–111 g) were smaller due to limited sample availability. Total food intake and meal completion time were recorded. At the end of each day, cats received an additional portion of their usual diet in the group housing area to compensate for reduced second meal portions and potential poor acceptance. Data were evaluated descriptively without statistical analysis. The results were used to select subjects showing excellent acceptance and higher intake for inclusion in the digestibility trial, maintaining equal sex distribution.

### 2.4 Digestibility trial

A double-blind, cross-over digestibility trial was conducted with eight cats (4 neutered males, 4 spayed females; age: 2 year; body weight: 3.3–5.0 kg; body condition score: 4–6/9; normal muscle condition) randomly allocated by sex into two groups. Each group received either the test or control diet during the first period, and switched diet in the second period. Each period lasted at least 12 days, comprising a 7–12-day dietary adaptation phase, the longer duration being due to an unforeseen rescheduling of the start of fecal collection, followed by 5 days of fecal collection. Food portions were calculated based on historical data on cat’s maintenance energy requirement. Adaptation phases allowed diet acclimation and adjustment of intake to maintain body weight (BW). Portions were fixed starting 2 days prior to the collection phase and remained constant throughout the collection period. Food consumption and leftovers were recorded after each meal. Cats were monitored daily for adverse effects, while BW, body condition score (BCS), and muscle condition score (MCS) were assessed weekly. All feces were collected, weighed, scored [1–7 scale; fecal scoring chart, Purina Pro Plan Veterinary Diets [Nestlé Purina] (36)], and stored at −20 °C. Diet samples and pooled fecal samples per cat per diet were analyzed for proximate composition (moisture, ash, crude protein, crude fat, crude fiber, insoluble ash) by a certified laboratory. Dry matter and ash were determined by drying to a constant weight at 103 °C and combustion at 550 °C, respectively. Nitrogen was determined by the Kjeldahl method (37) with crude protein calculated as N × 6·25, and crude fat was determined by pre-hydrolysis according to the Soxhlet method using diethyl ether (38). Crude fiber was analyzed by acid-alkali digestion using the Ankom device (39). Analyses were performed in duplicate, with the average as final result, and results were accepted if the coefficient of variance was <5%. Apparent digestibility was calculated using the indicator method as per FEDIAF guidelines (34), using the following formula:


$$\begin{array}{l} \text{Apparent digestibility of}\;a\;\text{nutrient}\\ =(1-\frac{\mathrm{AI}A_{\text{feed}}x\;\text{Nutrien}t_{\text{feces}}}{\mathrm{AI}A_{\text{feces}}x\;\text{Nutrien}t_{\text{feed}}})\times 100\% \end{array}$$

Where, $\mathrm{AI}A_{\text{feed}/\text{feces}}$ is the acid-insoluble ash in feed or feces and $\;\text{Nutrien}t_{\text{feces}/\text{feed}}$ is the nutrient content in feces or feed.

Nitrogen-free extract (NFE, % DM) was calculated as:


$$\mathrm{NFE}\;(\%\mathrm{DM})=[100-(\begin{array}{l} \%\text{crude protein}+\%\text{crude}\;\mathrm{fat}\\ +\%\text{crude}\;\mathrm{ash}+\%\text{crude fiber} \end{array})]$$

For crude fibre content, which was below the detection limit (<0.5 g/100 g fresh matter), a value of 0.1 g/100 g was used.

Metabolizable energy (ME, kJ/100 g DM) was estimated using the following equation:


$$\begin{array}{l} \text{Metabolizable energy}\;(\mathrm{KJ})=(15\;x\%\text{crude protein})\\ +(36\;x\%\text{crude}\;\mathrm{fat})+(15\;x\%\mathrm{NFE}) \end{array}$$

Food intake and BW data from both adaptation and collection phases were used to calculate average leftovers (%) and BW variation (%). The percentage of food leftovers was determined based on the ratio between the total weight of leftovers and the total weight of food offered during the trial. The percentage change in body weight for each period was calculated as the difference between the initial (day 3) and final (last day) weights, expressed relative to the initial weight.

All statistical analyses were performed using SPSS Analysis Software (IBM SPSS Statistics for Windows, Version 29.0.2.0 Armonk, NY). A generalized linear mixed model (GLMM) with diet, period and their interaction as fixed effects was applied to seven dependent variables: apparent digestibility of DM, ME, crude protein, crude fat, and NFE; leftovers (%); and BW variation (%). “Cat” was treated as a repeated subject with compound symmetry covariance. Normality was assessed via Kolmogorov–Smirnov and Shapiro–Wilk tests. All variables were normal except ‘leftovers’, which were right-skewed and analyzed using GLMM with gamma distribution and log link. *p*-values were adjusted using Benjamini-Hochberg FDR correction. Data on fecal scores, BCS and MCS were evaluated descriptively without statistical analysis.

## 3 Results

### 3.1 Acceptance test

The acceptance test demonstrated satisfactory overall acceptance of both diets. All cats accepted the control diet, with complete consumption observed in 17 of 20 meals, and at least 75% of the portion, based on food weight measurements, in the remaining meals. The test diet was well accepted by 9 out of 10 cats, with complete consumption in 17 of 18 meals, and 94% consumed in the remaining meal. However, one cat consumed only a small amount and subsequently refused the test diet entirely. Based on these observations, this individual was excluded from participation in the digestibility trial.

### 3.2 Diet composition

Proximate analysis of both diets confirmed a similar nutritional composition. Both diets had a high protein (above 50% DM), high fat (21–22% DM), low NFE (17–18% DM) and low fiber (<0.5% DM) content (Table 2).

**Table 2:** Results proximate analysis control and test diet.

Nutrient g/100 g DM	CO diet	CM diet
Crude protein	50.4	51.6
Crude fat	21.3	21.7
Crude Ash	9.6	8.9
Insoluble ash	1.7	1.8
Crude fiber*	<0.5	<0.5
NFE	18.1	17.2
ME kJ/100 g DM	1793.3	1813.6
Moisture	84.7	84.4

CO, conventional meat (control diet); CM, cultivated meat (test diet). *below detection limit expressed as g/100g fresh matter.

### 3.3 Digestibility test

Apparent digestibility data of crude fat, crude protein, NFE, metabolizable energy and dry matter are shown in Table 3. Protein digestibility was slightly but significantly higher in the control diet compared to the test diet [85.3 ± 1.6 vs. 83.9 ± 1.2%; *F*(1,12) = 8.181, *p* = 0.033]. No significant effect for all other digestibility variables was observed. Detailed digestibility data are available in Supplementary Tables 1, 2. Food intake data revealed a significantly lower percentage of leftovers for the test diet compared to the control diet [2.4 ± 3.3% vs. 8.5 ± 7.8%; *F*(1,12) = 10.311, *p* = 0.025]. Body weight variations were consistent with food intake observations, with significantly greater weight loss observed during consumption of the control diet [+0.8 ± 2.8 vs. −1.2 ± 1.9%; *F*(1,12) = 18.401, *p* = 0.007] and in the first experimental period [−1.4 ± 1.7 vs. +1.0 ± 2.8; F(1,12) = 26.137, *p* < 0.007]. Based on BW monitoring in the first period, food portions were slightly increased for most cats in the second adaptation period without substantial differences between groups. A summary of intake and BW data is presented in Table 4, full data are available in Supplementary Tables 3, 4. BCS and MCS remained stable throughout the trial. Fecal consistency remained generally optimal for both diets. For the control diet, 22 of 23 samples scored ideal (2 or 3), and all samples were within the range of 2–4. For the test diet, 21 of 25 samples were ideal, and all scores fell within the range of 1–5. Three cats exhibited a single mixed-consistency episode with each diet, where part of the sample was ideal and part liquid; these were isolated and did not affect health. No persistent clinical signs occurred. One cat vomited once on day 1 while receiving the test diet. A minor skin lesion was observed in another cat during adaptation to the test diet; it resolved spontaneously within a few days without dietary change and was considered unlikely to be diet-related.

**Table 3:** Overview of apparent digestibility (%) results.

%	CM diet	CO diet	Diet effect *P*-values	Period effect *p*-values	Diet*period interaction *p*-values
AD crude protein	83.9 ± 1.2	85.3 ± 1.6	**0.033**	0.553	1.432
AD crude fat	92.6 ± 1.1	93.3 ± 1.3	0.089	0.846	1.015
AD NFE	75.1 ± 1.3	73.5 ± 2.1	0.115	0.797	0.998
AD ME	86.4 ± 0.7	86.9 ± 1.1	0.207	0.773	0.961
AD DM	79.3 ± 0.7	79.7 ± 1.3	0.464	0.735	1.116

On the left, means and standard deviations of the apparent digestibility for each diet; on the right, statistically significant *p*-values shown in bold. CM, cultivated meat; CO, conventional meat; AD, apparent digestibility; NFE, nitrogen-free extract; ME, metabolizable energy; DM, dry matter.

**Table 4:** Data on leftovers and body weight variation (%).

%	CM diet	CO diet	Period 1	Period 2	Diet effect *P*-values	Period effect *P*-values	Diet*period interaction *P*-values
Leftovers	2.4 ± 3.3	8.5 ± 7.8	5.9 ± 6.6	5.0 ± 6.9	**0.025**	0.567	1.141
Variation BW	+0.8 ± 2.8	−1.2 ± 1.9	−1.4 ± 1.7	+1.0 ± 2.8	**0.007**	**<0.007**	2.639

On the left, means and standard deviations for each diet and period; on the right, significant *p*-values shown in bold. CM, cultivated meat; CO, conventional meat.

## 4 Discussion

This study provided the first *in vivo* assessment of apparent digestibility of a cultivated cell biomass incorporated into a complete feline diet. To date, published data on digestibility of CM are limited and come from studies on CM for human consumption (40, 41), using a static *in vitro* digestion model which simulates human gastrointestinal conditions. Such findings cannot be extrapolated to cats. Moreover, similar in vitro methods applied in pet food research have been shown to overestimate nutrient digestibility (42), making *in vivo* feeding trials the golden standard for evaluating nutrient digestibility in companion animals. The results of the present study, derived from emerging cellular agriculture technologies and involving cells from a species not commonly used in pet food or the food system, provided unique insights into the nutritional performance of CM for animal nutrition.

Cultivated meat has recently gained attention as a potential sustainable protein source for pet food. Although not yet commercially available, its future adoption appears likely. Current evidence shows that CM can approximate the nutritional and physicochemical properties of conventional meat, though marked variability remains due to differences in cell type, scaffold materials, and cultivation methods (43–45). This study supported nutritional similarity, demonstrating that a feline diet incorporating CM can match the composition of a conventional meat-based diet. Among these parameters, moisture content emerges as one of the most divergent features. While conventional meat typically contains 54–75% moisture (46), CM exhibits higher values (75–90%) (24). Similarly, the cultivated cell biomass tested in this study had a considerably higher water content (81.5%) than meat, which required additional water in the control diet to achieve comparable composition, resulting in more liquid products. The control and test diets contained 84.4 and 84.7% moisture respectively, placing them at the upper end of the reported range for feline wet foods (60–87%) (47) whose typical mean is 76% (48). This characteristic may offer advantages for formulations targeting urinary health, constipation or weight management. Cats have a relatively low thirst drive and, unlike dogs, do not fully compensate for a low moisture in their diet, leading to reduced total water intake and a predisposition to suboptimal hydration (49). Increasing dietary moisture promotes urine dilution, fecal water content, and satiety, thereby reducing the risk of lower urinary tract disease and constipation, and facilitating weight loss (50–53). Conversely, higher moisture lowers nutrient and energy density on an as-fed basis, requiring compensatory formulation adjustments and potentially higher inclusion of CM compared to conventional. In dry diets, meeting the low moisture limits for microbial stability (≤10–12%) (34) would require longer dehydration steps of the CM, increasing processing energy demand. In addition to nutritional aspects, economic feasibility is an important consideration for real-world application of CM. At the inclusion level tested, the production cost of the overall diet is comparable to that of commercial super-premium pet foods, indicating potential economic viability.

### 4.1 Macronutrient digestibility

Average apparent digestibility coefficients in commercial pet foods, approximately ≥80% for crude protein, 90% for crude fat, and 85% for NFE, formed the basis of the modified Atwater equation, a widely accepted method for energy calculation in pet food (54). Compared to this values, both diets demonstrated optimal fat and protein digestibility, with values above this standard, but less optimal NFE digestibility (73.5% in the control, 75.1% in the test diet). Nonetheless, these estimates are indicative and may vary depending on factors such as ingredient composition, raw material quality, and processing conditions. The NFE digestibility observed in this study is consistent with previous findings reporting lower values for canned cat food compared to dry food (73.3 ± 20.7% vs. 89.1 ± 5.5%), based on analysis of 227 studies involving both commercial and non-commercial feline diets (48). Such difference is primarily influenced by processing conditions, as thermal treatments, particularly extrusion, are known to enhance starch digestibility (55). In contrast, when compared to data from feline dry diets formulated with peas, the same starch source included in both study diets, significantly higher NFE digestibility has been reported. De-Oliveira et al. (56) found that peas incorporated into an extruded dry diet achieved an NFE apparent digestibility of 96.3%, compared to 73.5 and 75.1% in the present study. This highlights the potential for pea to be highly digestible when properly processed through extrusion, underscoring the critical role of manufacturing techniques in optimizing nutrient digestibility. Importantly, NFE digestibility results obtained in the present study reflected the choice and processing of the starch source and were unrelated to the protein sources evaluated in this study. Additionally, the fat apparent digestibility values reported by Hall et al. (48) for wet feline diets (88.2% ± 6.5%) are also aligned with those observed in the present study. To the authors’ knowledge, data on protein digestibility in wet feline diets containing chicken breast meat are lacking, as this ingredient is uncommon in commercial formulations, limiting direct comparisons. However, Kerr et al. (57) reported an apparent crude protein digestibility of 84.1% for canned feline diets based on chicken meat and poultry by-products, a value comparable to those observed for both control and test diets in the present study. Although the difference in apparent protein digestibility between the two diets reached statistical significance, its magnitude was modest (1.4 percentage points). Such a limited difference should be interpreted cautiously, as statistical significance does not necessarily imply clinical significance. Moreover, as previously discussed, the protein digestibility of the test diet is comparable to those reported for commercial chicken-based canned food (83.9% vs. 84.1%) (57). It is also worth noting that the present study employed a high benchmark ingredient (human-grade chicken breast) as the control, which is not typically used in standard pet food formulations. In summary, data confirm a difference in apparent protein digestibility, although its magnitude may be of limited clinical relevance under typical feeding conditions.

### 4.2 Body weight and food intake

The significant period effect on body weight likely reflects inaccuracies in initial dietary energy density estimates, based on a different batch and predictive models, such as modified Atwater and National Research Council (NRC) equations, which offer only moderate accuracy for ME in feline wet diets (34). This was mitigated in the second period by adjusting food portions based on body weight monitoring in the first period. When comparing acceptance and intake between diets, differences in texture and inclusion of palatability enhancers must be considered. Although the diets were similar, differences in texture were present; these may have influenced palatability and intake, given that texture also plays a role in cats’ food preferences (33). The control diet contained 10 g/kg of palatability enhancers versus 1.5 g/kg in the test diet, a difference introduced after observing poor acceptance of the control diet during development, necessitating a higher inclusion rate to ensure adequate intake. Interestingly, despite the lower palatability enhancer inclusion, the test diet exerted a significant positive effect on food intake. This also explains the positive effect of the test diet in maintaining stable body weight. These findings are particularly noteworthy given the expectation that higher palatability enhancer levels would promote greater consumption. The observed outcome may suggest a higher intrinsic palatability of the cultivated hamster cells compared to chicken breast meat. However, this hypothesis warrants further investigation through a dedicated palatability study employing standardized two-bowl or preference testing protocols.

### 4.3 Limitations of the study

Apparent total tract digestibility provides a practical, non-invasive estimate of overall nutrient digestibility under *in vivo* feeding conditions in the target species. However, AD does not assess protein quality or the bioavailability of individual indispensable amino acids, and should therefore be interpreted accordingly. The species difference between the ingredients (traditional chicken meat vs. cultivated hamster cells) may have played a role in influencing aroma profile and digestibility. Therefore, the results of this study must be interpreted as a comparative performance between a novel ingredient and a traditional high benchmark, rather than as a direct comparison between conventional and cultivated meat. The trial periods were relatively short (12–17 days), which may not fully capture long-term diet effects on acceptance, food intake and clinical signs. Also, cats were selected for the digestibility trial based on their performance in a short-term acceptance test; this may have introduced a bias toward individuals with higher baseline palatability responses, potentially limiting intake variability and masking diet-related differences. Future studies should evaluate the inclusion of cultivated meat ingredients in larger animal populations and over longer feeding periods to assess long-term nutritional adequacy, health outcomes, and palatability.

## 5 Conclusion

The present study showed that the inclusion of the cultivated hamster cell biomass tested into a complete feline diet, was well accepted and digested by cats. Further research is warranted to explore a possible positive impact on palatability and to assess long-term health while feeding this novel ingredient to cats.

## Data Availability

The original contributions presented in the study are included in the article/Supplementary material, further inquiries can be directed to the corresponding author.
