# Supplementary material for: Can cultivated hamster cells compete with chicken meat? Insights on acceptance and digestibility in domestic cats
Source: Front Vet Sci. 2026 May 21;13:1781530. doi: 10.3389/fvets.2026.1781530 (PMC13235436; doi:10.3389/fvets.2026.1781530)
Supplement: Supplementary file 1 [file Data_Sheet_1.PDF]

Supplementary table 1

Results analysis food and fecal samples for each period used to calculate apparent digestibility - CONTROL DIET (CO, conventional chicken meat)

		FRESH MATTER										DRY MATTER						
Lab ID	Cat/diet ID	Moisture (%)	g dry matter/100 g fresh matter	g ash/100 g fresh matter	g crude fibre/100 g fresh matter	g fat/100 g fresh matter	g protein/100 g fresh matter	g celite/100 g fresh matter	g NFE/100g FM	ME KJ/100g		g ash/100 g dry matter	g crude fiber/100 g dry matter	g fat/100 g dry matter	g protein/100 g dry matter	g celite/100 g dry matter	g NFE/100g DM	ME KJ/100g DM
17	Diet 1	84,69	15,31	1,46	0,10	3,26	7,72	0,26	2,77	274,52		9,56	0,65	21,26	50,41	1,72	18,11	1,793,30
	**Digestibility trial period 1** (fecal collection phase - 120 h)																	
4	cat 1	69,64	30,36	8,22	1,97	2,21	10,60	2,56	7,37	349,05		27,06	6,48	7,28	34,92	8,43	24,26	1,149,75
2	cat 2	72,07	27,93	6,96	2,49	2,43	10,62	2,22	5,44	328,21		24,91	8,91	8,69	38,02	7,93	19,46	1,175,03
5	cat 3	70,45	29,55	8,27	1,80	1,55	10,55	2,64	7,37	324,83		27,99	6,10	5,26	35,70	8,95	24,95	1,099,20
7	cat 4	76,25	23,75	6,31	1,69	1,72	8,25	1,96	5,77	272,18		26,57	7,13	7,23	34,75	8,27	24,31	1,146,12
	**Digestibility trial period 2** (fecal collection phase - 120 h)																	
16	cat 5	72,41	27,59	7,30	1,72	2,11	9,55	2,21	6,92	322,86		26,46	6,22	7,63	34,61	8,02	25,07	1,170,09
14	cat 6	73,82	26,18	7,03	1,58	1,75	9,39	2,52	6,44	300,27		26,86	6,02	6,67	35,86	9,63	24,59	1,146,87
11	cat 7	70,86	29,14	7,76	0,44	1,97	12,55	2,43	6,42	355,43		26,64	1,51	6,76	43,05	8,33	22,04	1,219,80
9	cat 8	74,54	25,46	6,79	1,69	1,54	9,06	2,21	6,38	287,07		26,67	6,64	6,06	35,58	8,68	25,04	1,127,57

Supplementary table 2

Results analysis food and fecal samples for each period used to calculate apparent digestibility - TEST DIET (CM, cultivated meat)

		FRESH MATTER										DRY MATTER						
Lab ID	Cat/diet ID	Moisture (%)	g dry matter/100 g fresh matter	g ash/100 g fresh matter	g crude fibre/100 g fresh matter	g fat/100 g fresh matter	g protein/100 g fresh matter	g celite/100 g fresh matter	g NFE/100g FM	ME KJ/100g		g ash/100 g dry matter	g crude fiber/100 g dry matter	g fat/100 g dry matter	g protein/100 g dry matter	g celite/100 g dry matter	g NFE/100g DM	ME KJ/100g DM
18	Diet 2	84,41	15,59	1,38	0,10	3,39	8,03	0,28	2,68	282,65		8,86	0,64	21,72	51,55	1,77	17,23	1,813,59
	**Digestibility trial period 1** (fecal collection phase - 120 h)																	
8	cat 5	69,29	30,71	7,90	1,94	2,62	11,82	2,69	6,43	368,08		25,73	6,31	8,54	38,49	8,77	20,93	1,198,74
6	cat 6	74,60	25,40	6,54	1,58	1,87	10,13	2,09	5,29	298,48		25,75	6,21	7,35	39,87	8,21	20,83	1,174,96
3	cat 7	68,02	31,98	7,94	1,24	2,14	14,39	2,63	6,27	386,89		24,82	3,88	6,68	44,99	8,23	19,62	1,209,81
1	cat 8	75,44	24,56	6,13	1,93	1,79	9,96	2,05	4,74	285,08		24,98	7,87	7,30	40,54	8,34	19,31	1,160,67
	**Digestibility trial period 2** (fecal collection phase - 120 h)																	
12	cat 1	69,29	30,71	8,07	1,58	2,52	12,09	2,66	6,45	368,76		26,28	5,15	8,19	39,37	8,67	21,01	1,200,67
10	cat 2	69,19	30,81	7,75	2,11	2,65	12,12	2,67	6,18	369,90		25,15	6,86	8,61	39,34	8,65	20,04	1,200,60
13	cat 3	69,81	30,19	8,16	1,66	1,87	12,08	2,73	6,42	344,91		27,02	5,49	6,20	40,02	9,04	21,26	1,142,51
15	cat 4	74,00	26,00	6,52	1,09	2,50	9,94	2,22	5,94	328,26		25,07	4,21	9,62	38,25	8,56	22,85	1,262,79
