# Supplementary material for: Can cultivated hamster cells compete with chicken meat? Insights on acceptance and digestibility in domestic cats
Source: Front Vet Sci. 2026 May 21;13:1781530. doi: 10.3389/fvets.2026.1781530 (PMC13235436; doi:10.3389/fvets.2026.1781530)
Supplement: Supplementary file 2 [file Data_Sheet_2.PDF]

Supplementary table 3

Leftovers and Food intake monitoring, total period (adaptation + collection phase)

	Control diet (CO)					Test diet (CM)				
	total amount fed (g/period)	average g/meal	leftovers (g/period)	% leftovers	% intake	total amount fed (g/period)	average g/meal	leftovers (g/period)	% leftovers	% intake
	**Period 1** (12 days)					**Period 2** (17 days)				
**Cat 1**	2976	124	108	3,6	96,4	4160	130	129	3,1	96,9
**Cat 2**	4440	185	312	7,0	93,0	5846	183	93	1,6	98,4
**Cat 3**	3384	141	98	2,9	97,1	5030	157	0	0,0	100,0
**Cat 4**	4224	176	852	20,2	79,8	5846	183	49	0,8	99,2
	**Period 2** (17 days)					**Period 1** (12 days)				
**Cat 5**	4475	140	647	14,4	85,6	3168	132	68	2,1	97,9
**Cat 6**	5606	175	60	1,1	98,9	4032	168	0	0,0	100,0
**Cat 7**	4425	138	777	17,6	82,4	3384	141	340	10,0	90,0
**Cat 8**	5554	174	61	1,1	98,9	4224	176	54	1,3	98,7
**Mean**	4386	157	364	8,5	91,5	4461	159	92	2,4	97,6
**SD**	917	23	341	7,8	7,8	1023	22	109	3,3	3,3
**Min**	2976	124	60	1,1	79,8	3168	130	0	0,0	90,0
**Max**	5606	185	852	20,2	98,9	5846	183	340	10,0	100,0

Supplementary table 4

Body weight monitoring - 3rd and last day of each period

		**Period 1-CTRL (CO)**		**period 2- TEST (CM)**	
	(kg)	day 3	day 12	day 3	day 17
Cat 1	BW	4,10	4,01	4,11	4,11
Cat 2	BW	3,76	3,73	3,77	3,91
Cat 3	BW	3,71	3,62	3,56	3,64
Cat 4	BW	4,13	4,08	4,10	4,28
		**Period 1-TEST (CM)**		**period 2 - CTRL (CO)**	
		day 3	day 12	day 3	day 17
Cat 5	BW	3,47	3,42	3,41	3,33
Cat 6	BW	4,88	4,86	4,86	4,85
Cat 7	BW	3,41	3,27	3,41	3,31
Cat 8	BW	4,11	4,18	4,11	4,23

CM= cultivated meat

CO= conventional chicken meat
